# Smaller household size and higher prevalence of serious psychological distress in younger people and never-married people: a nationwide cross-sectional survey in Japan

**DOI:** 10.3389/fpubh.2024.1292371

**Published:** 2024-03-11

**Authors:** Kimiko Tomioka, Midori Shima, Keigo Saeki

**Affiliations:** Nara Prefectural Health Research Center, Nara Medical University, Kashihara, Japan

**Keywords:** household size, living alone, serious psychological distress, Kessler 6-item psychological distress scale, national representative survey, cross-sectional studies

## Abstract

**Background:**

Small-member households are increasing worldwide. However, most previous studies have focused on older people and living alone. Using the latest national survey data, we investigated a dose–response relationship between household size and serious psychological distress (SPD).

**Methods:**

We analyzed data from the 2019 Comprehensive Survey of Living Conditions in Japan. The study participants were 405,560 community-dwelling adults aged 20 or older. Household size was classified into 5 or more, 3 or 4, two, and one (i.e., living alone). SPD was defined as ≥13 points based on the Kessler 6-item Psychological Distress Scale. We used multivariable logistic regressions and included age, education, equivalent household expenditures, housing tenure, employment contract, smoking, and illness under treatment as covariates.

**Results:**

After stratified analyses by age and gender, a dose–response relationship between smaller household size and more common SPD was significant for younger, but not for older people (*p*-trend was <0.001 in men aged 20–59 and women aged 20–39). After stratified analyses by gender and marital status, a dose–response relationship was significant only for the never-married group in both genders (*p*-trend was <0.001 in never-married men and women).

**Conclusion:**

Smaller households were associated with higher prevalence of SPD in younger adults and in never-married individuals, regardless of gender. Our findings suggest a need to focus on younger people and never-married people to reduce the mental health risks due to small household sizes.

## Introduction

The percentage of single-person households is increasing worldwide (1). In Japan, the proportion of one-person households increased from 20.8% in 1985 to 38.1% in 2020 (2). The increase in single-person households can be attributed to a variety of reasons, including late marriage, an increase in the number of divorced people, and an increase in the number of people who have never been married (3). For some people, this may be a conscious decision based on changing societal attitudes towards marriage. However, others have simply given up on getting married and starting a family because they were unable to find full-time employment (3). An increase in the number of old people living alone leads to an increase in “*kodokushi*” in which people living alone die at home and are found after death (4), which has become a social problem in Japan.

Individuals living alone can be vulnerable, not only in their financial situation, but also in their physical and mental state (5) and have been reported to have a higher risk of mortality than those living with others (6). In this association, social isolation and emotional loneliness may adversely affect mental health and increase mortality risk (7, 8). Nonetheless, many of the studies on the relationship between living alone and mental health have focused on older people (9–12), and there have been few studies on the general population (8, 13).

In Japan, not only the number of one-person households but also the number of small-member households is increasing (1). For example, the percentage of one- or two-person households was around 20% in 1955, but exceeded 40% in 1989, reaching 60.8% in the latest 2019 data (14). In Japan, where familism is stronger and social participation is less active than in Western countries, the shift to nuclear families may lead to an increase in the number of people who become socially isolated (15). Due to the prolonged impact of the spread of the Coronavirus disease 2019 (COVID-19), the problems of loneliness and isolation inherent in society have become apparent and become a serious social problem. In May 2023, the Act on Promotion of Policy for Loneliness and Isolation was passed that clearly states that loneliness and isolation measures are necessary not only for older people but also for all generations (16).

A household is defined as one individual or group of people who usually live together and share livelihood. Household members are often composed of family members, but may also include people who have no kin relationship. Furthermore, in modern society, the number of people who are not married but living together with a partner (i.e., common-law couples) is increasing. The true extent of common-law relationships in Japan is unknown, but according to several surveys conducted in 2021, common-law couples accounted for about 2% of survey respondents aged 20 and older (3). Amid changes in family and marriage circumstances, therefore, it is more appropriate to focus on the number of household members (i.e., household size) rather than family size. Households create a social environment that is critical to maintaining the health of the members who live in them, through enabling household members to encounter this environment on a daily basis, perform social roles, and enjoy social relationships (17). Previous ecological studies have shown that household size is negatively correlated with cancer incidence (18) and dementia mortality (17). Large household size may offer more subjective happiness and more life satisfaction to household members (19, 20), which in turn may prevent psychological distress. Support from household members is an important source of social support for each household member, and enriches their social ties (21). However, the effects of social ties on mental health differ by gender. A previous study suggests that household size is a good estimator of demands from household members (22). Because women are responsible for housework and childcare, it is possible that the larger the household size, the greater the mental burden on women (21, 22). Furthermore, men are more dependent on support from their spouses than women (21). These previous studies suggest that men may derive more mental health benefits from large households than women. In addition, the effects of living alone or living with others on mental health may differ depending on age and marital status as well as gender (6, 9, 23–25). However, to our knowledge, no studies have examined demographic differences in an association between household size and mental health. By examining the associations between household size and serious psychological distress (SPD) by age, gender, and marital status, we can provide information that may support efforts to effectively deal with loneliness and isolation, which is a growing concern in Japan.

We formed the following research hypothesis: (1) Small household size is associated with SPD; (2) If there is a possible association between small households and SPD, a dose–response relationship may be seen. Therefore, the purpose of this study is to examine a dose–response relationship between household size and SPD by age, gender, and marital status, using the latest publicly available data from a large national cross-sectional survey in Japan.

## Materials and methods

### Data source

We analyzed the 2019 survey data from the Comprehensive Survey of Living Conditions (CSLC) (26), a large-scale nationwide survey by the Ministry of Health, Labour and Welfare. Details on the CSLC are presented in Supplementary Text 1. The number of households surveyed in the CSLC was 301,334, and the number of valid responses was 217,179 (valid response rate of 72.1%).

### Study participants

**Figure 1:**
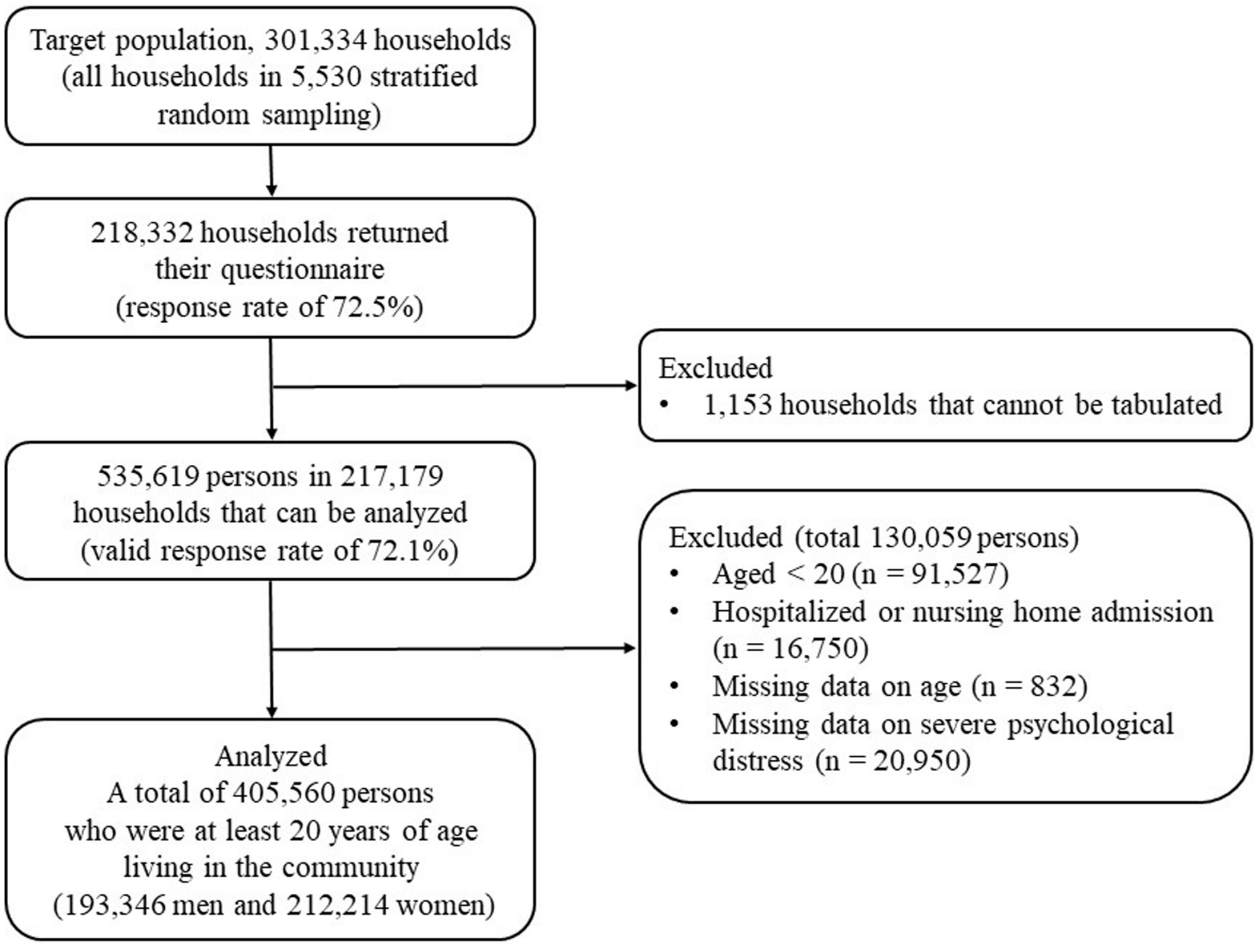
Selection of study participants.

The aim of this study was to examine the association between household size and SPD among Japanese adults living in the community. Therefore, our study focused on community residents aged 20 or older (excluding those who were hospitalized or in nursing care facilities), and the sample in this age range included 427,342 subjects. Excluding 21,782 individuals for whom age or SPD information was missing, our final study population was 405,560 (193,346 men and 212,214 women), representing 94.9% of the sample. Details of the selection of study participants are shown in Figure 1.

### Measures

#### Household size

The question on household status is presented in Supplementary Table S1. In this study, we defined the number of household members as household size. Then, household size was classified into 5 or more, 3 or 4, two, and one (i.e., living alone).

#### Serious psychological distress

The Japanese version of the Kessler 6-item Psychological Distress Scale (K6) was used to assess SPD (27). The K6 has been used in the CSLC since 2007 to assess the state of mental health of the public (26). The K6 is a psychological scale developed for the purpose of screening the mental health of adults in the general population (28). The K6 questionnaire is presented in Supplementary Table S2. The K6 has been reported to have sufficient reliability and validity (28, 29). In accordance with a cut-off point of 13 or higher that has been demonstrated to be sufficiently effective to identify community residents with severe mental disorders (29), previous studies have defined a K6 score of 13 or higher as SPD (30–32). In this study, participants with a K6 score of 13 or higher were defined as those with SPD, and those with a K6 score of 12 or lower were defined as those without SPD.

#### Covariates

Previous studies on the association of living alone and/or living arrangements with mental health have identified socio-economic status, smoking, and chronic physical conditions as important confounding factors (5–13). Therefore, in this study, the following variables were used as covariates: marital status, education, equivalent household expenditures (EHE), employment contract, housing tenure, smoking status, and illness under treatment. A category entitled “missing” was used for values that were missing in responses to questions on the covariates. Details on the covariates are presented in Supplementary Text 2. Variance inflation factor values were less than 2.0 for all variables, indicating the absence of multicollinearity problems.

### Statistical analyses

Means, medians, and proportions between men and women were compared using the *t*-test, the Mann–Whitney test, and the Chi-squared test.

We used multivariable logistic regression models to calculate the adjusted odds ratio (AOR) with 95% confidence interval (CI) for SPD. The dependent variable was SPD (a total K6 score of ≥13 points). The independent variable was household size (5 or more, 3 or 4, two, and one). A dose–response relationship between household size and SPD was tested using logistic regression with a continuous variable of household size (1 = living alone, 2 = two-member household, 3 = household size of three or four, and 4 = household size of five or more). First, we performed stratified analyses by age and gender, and entered all covariates simultaneously. Age was classified into 4 groups: aged 20–39 years, aged 40–59 years, aged 60–74 years, and aged 75 and older. Next, we conducted additional analyses by gender and marital status to examine whether the association between household size and SPD differed by gender and marital status. Common-law marriage was defined as married, and marital status was classified into 3 groups: married, never-married, and widowed/divorced. The significance level was set at *p* < 0.05. Analyses were performed using the IBM SPSS Statistics Ver. 27 for Windows (Armonk, New York, NY, United States).

### Ethics approval statement

This study was approved by the Nara Medical University Ethics Committee (approval number 3325), and received approval from the Ministry of Health, Labour and Welfare to use questionnaire information of the Comprehensive Survey of Living Conditions for research purposes (approval number 2022-0420-1). Participant consent was not obtained because all data were fully anonymized before we accessed them.

## Results

**Figure 2:**
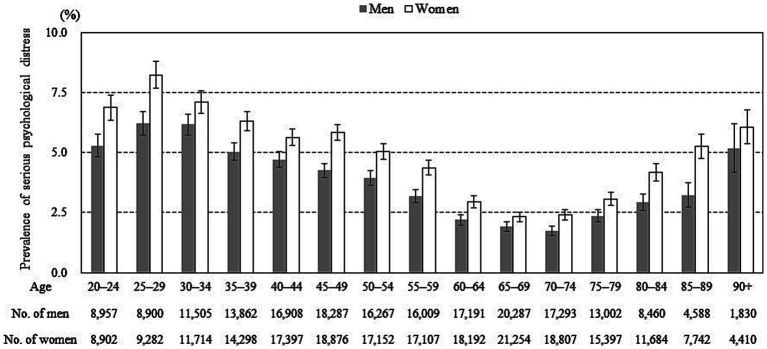
Prevalence of serious psychological distress according to age and gender. Error bars display 95% confidence intervals.

Among study participants, the prevalence of people with SPD was 3.6% in men and 4.7% in women, showing a significant gender difference (*p* < 0.001). The prevalence of SPD by age and gender (Figure 2) was highest in people aged 25–29, decreased in people aged 30–60, was lowest in people aged 65–74, and increased again after age 75. These age-related differences showed the same trend for both genders. In addition, the prevalence of SPD was higher in women than in men across all age groups.

For marital status by age and gender (Supplementary Figure S1), regardless of gender, the percentage of never-married people decreased with age and the percentage of divorced or widowed people increased with age. The age group with the lowest percentage of married people was younger people aged 20–39 for men, but older people aged 75 or older for women. For basic characteristics of participants by gender (Table 1), men were more likely to be married, to be highly educated, to have more EHE spending, to be regular employees, to be current smokers, and to be treated for chronic medical conditions, while women were more likely to be older, to be owner-occupiers, and to live alone.

**Table 1:** Basic characteristics of analyzed participants by gender.

	Men	Women	*p*-value
	(*n* = 193,346)	(*n* = 212,214)	
Age, years, mean (SD)	54.7 (17.6)	56.4 (18.3)	<0.001^a^
Marital status: married (%)	69.5%	63.0%	<0.001^b^
Education: ≥16 years (%)	27.7%	13.2%	<0.001^b^
Equivalent household expenditures: median (IQR)	13.4 (7.7)	13.0 (7.3)	<0.001^c^
Employment contract: regular employees (%)	43.8%	20.7%	<0.001^b^
Housing tenure: owner-occupiers (%)	78.8%	79.5%	<0.001^b^
Smoking status: current smokers (%)	29.8%	8.5%	<0.001^b^
People with chronic medical conditions (%)	20.4%	16.6%	<0.001^b^
Household size			
Mean (SD)	2.99 (1.43)	2.98 (1.44)	0.017^a^
One (people living alone) (%)	12.3%	12.7%	0.002^b^
Kessler 6-item Psychological Distress Scale score			
Median (IQR)	1.00 (5.00)	2.00 (6.00)	<0.001^c^
≥13 points (people with SPD) (%)	3.6%	4.7%	<0.001^b^

IQR, interquartile range; SD, standard deviation; SPD, serious psychological distress. The unit of equivalent household expenditures is ten thousand Japanese yen per month.

a Means between men and women were compared using the *t*-test.

b Proportions between men and women were compared using the Chi-squared test.

c Medians between men and women were compared using the Mann–Whitney test.

For the results of the gender stratification analysis (Supplementary Table S3), a dose–response relationship indicating the smaller the household size, the greater the number of people with SPD, was significant for men, but not for women (*P* for trend was <0.001 in men, and 0.146 in women). For variables other than household size, both men and women showed the same tendency. SPD was significantly more common among unmarried individuals, younger people, people with low education, the non-working group, people without homeownership, people with a smoking history, and those with illness under treatment, while EHE was not associated with SPD, regardless of gender.

For the results of stratified analyses by age and gender (Table 2), a significant dose–response relationship between household size and SPD was observed in the younger age group of men aged 20–59 and women aged 20–39 but not in those in the older age groups (*P* for trend was <0.001 in men aged 20–39, men aged 40–59, and women aged 20–39). The results suggest that an association between smaller household size and more prevalent SPD is observed only in younger, but not in older people, regardless of gender. Furthermore, among women aged 60–74, those with household size of 3–4 or 2 people were significantly more likely to report SPD than those with 5 or more household members, but living alone was not associated with SPD. In contrast, for men aged 60–74, no relationship was found between household size and SPD. These results indicate that among women aged 60–74, the high prevalence of SPD is observed in people living with 2 to 4 household members, as opposed to those living alone.

**Table 2:** Adjusted odds ratio of household size for serious psychological distress by age and gender.

	Aged 20–39		Aged 40–59		Aged 60–74		Aged 75 or older	
	*N*	AOR (95% CI)	*N*	AOR (95% CI)	*N*	AOR (95% CI)	*N*	AOR (95% CI)
Men								
Household size								
≥5	8,363	1.00	10,660	1.00	4,292	1.00	2,784	1.00
3 or 4	24,215	1.09 (0.97–1.23)	35,545	1.02 (0.90–1.16)	17,910	1.05 (0.81–1.36)	7,292	1.09 (0.83–1.43)
Two	5,389	1.33 (1.13–1.56)^*^	13,875	1.17 (1.02–1.36)^*^	24,912	0.93 (0.72–1.20)	14,259	1.03 (0.80–1.34)
One	5,257	1.96 (1.64–2.34)^**^	7,391	1.52 (1.28–1.81)^**^	7,657	0.90 (0.66–1.24)	3,545	0.99 (0.70–1.39)
	*P* for trend <0.001		*P* for trend <0.001		*P* for trend = 0.159		*P* for trend = 0.737	
Women								
Household size								
≥5	9,322	1.00	10,940	1.00	4,868	1.00	3,790	1.00
3 or 4	25,447	0.98 (0.88–1.08)	37,923	0.95 (0.86–1.06)	17,376	1.42 (1.13–1.79)^*^	10,479	1.08 (0.90–1.30)
Two	5,994	1.40 (1.22–1.60)^**^	16,906	1.01 (0.90–1.14)	27,916	1.28 (1.02–1.60)^*^	14,383	0.97 (0.81–1.17)
One	3,433	1.78 (1.50–2.11)^**^	4,763	1.05 (0.89–1.23)	8,093	1.21 (0.92–1.59)	10,581	1.04 (0.85–1.27)
	*P* for trend <0.001		*P* for trend = 0.346		*P* for trend = 0.932		*P* for trend = 0.692	

AOR, adjusted odds ratio; CI, confidence interval. ^*^p < 0.05 and ^**^p < 0.001. AOR was adjusted for marital status, age, education, equivalent household expenditures, employment contract, housing tenure, smoking status, and illness under treatment.

For the results of additional stratified analyses by gender and marital status (Table 3), a significant association between smaller household size and more common SPD was found in never-married persons of both genders (*P* for trend was <0.001 in never-married persons of both genders). The results suggest that never-married persons living in smaller households are more likely to report higher levels of SPD than those in larger households, regardless of gender.

**Table 3:** Adjusted odds ratio of household size for serious psychological distress by gender and marital status.

	Married		Never-married		Widowed/divorced	
	*N*	AOR (95% CI)	*N*	AOR (95% CI)	*N*	AOR (95% CI)
Men						
Household size						
5 or more	20,611	1.00	4,581	1.00	907	1.00
3 or 4	61,919	1.00 (0.91–1.10)	19,986	1.16 (0.999–1.35)	3,057	1.68 (1.08–2.61)^*^
Two	48,299	0.97 (0.87–1.08)	7,025	1.46 (1.22–1.75)^**^	3,111	1.62 (1.04–2.54)^*^
One (living alone)	3,517	0.96 (0.77–1.20)	12,518	1.89 (1.58–2.26)^**^	7,815	1.74 (1.13–2.68)^*^
	*P* for trend = 0.477		*P* for trend <0.001		*P* for trend = 0.081	
Women						
Household size						
5 or more	20,893	1.00	3,975	1.00	4,052	1.00
3 or 4	62,961	0.95 (0.87–1.03)	16,206	1.02 (0.89–1.17)	12,058	1.31 (1.10–1.56)^*^
Two	48,690	0.93 (0.85–1.02)	5,057	1.37 (1.16–1.63)^**^	11,452	1.22 (1.01–1.46)^*^
One (living alone)	1,168	1.29 (0.98–1.69)	8,268	1.64 (1.37–1.96)^**^	17,434	1.07 (0.89–1.28)
	*P* for trend = 0.388		*P* for trend <0.001		*P* for trend = 0.104	

AOR, adjusted odds ratio; CI, confidence interval. ^*^p < 0.05 and ^**^p < 0.001. AOR was adjusted for age, education, equivalent household expenditures, employment contract, housing tenure, smoking status, and illness under treatment. Married includes common-law couple.

## Discussion

After stratified analyses by age and gender, a dose–response relationship, indicating the smaller the household size, the greater the number of people with SPD, was significant among men aged 59 or younger and among women aged 39 or younger. In stratified analyses by gender and marital status, a significant dose–response relationship was observed for both men and women who had never married. This study is the first to show that a dose–response relationship between smaller household size and more common SPD is found in younger people and in never-married individuals, and these dose–response relationships are consistent in both genders.

Regarding comparisons with previous studies, a systematic review and meta-analysis reported that living alone increased the risk of death only in younger people under 65 years but not in older people over 75 years (6). Although outcomes are different, this previous study suggests that the adverse health effects of living alone are greater for working-age people than for older people, supporting our findings. Previous research on the association between living alone and psychological distress found no association in both genders in a Korean study of persons aged 19 and over (13), an association among women and all ages in a Ghanaian study of persons aged 50 and over (33), and an association only in men in a Finnish study of persons aged 30 to 64 (24), with inconsistent gender differences and insufficient consideration of age differences. The different ages of study participants, the different patterns of people with psychological distress by age group, the different survey years, and the different cultural backgrounds may have caused the inconsistent results in the previous studies. By contrast, the present study used the 2019 national survey data with a high response rate among a randomly selected population. Therefore, our study can be generalized to Japanese households.

Regarding mechanisms, first, people who live with others may get more encouragement to stay healthy, and may have quicker access to medical services and first aid in case of illness (6). A prior study has reported that people living alone are more likely to be current smokers than people who live with others (5). The association between living alone and health is thought to be stronger in younger people than older people, because younger people tend to be less interested in their own health and less good at managing their own health (6). Second, regarding the adverse effects of living alone on mental health, the feeling of social isolation and loneliness is considered to be a psychosocial risk factor (8, 34). Moreover, not only living alone, but also the size of social networks has been taken as an indicator of social isolation (7). Social networks include both close relationships with family members, close relatives, and friends and spontaneous casual relationships in community, voluntary, and religious organizations. Because younger people have less informal personal and community ties than older people (6, 35), younger people living in smaller households often feel social isolation and loneliness, which may be associated with SPD. Because Japan has a public nursing care and monitoring system for older people (36, 37), older people who live alone or in a small household may be able to have connections with people other than their household members. Although the health effects of living alone have focused on older people, the results of this study suggest that the adverse effects of small household sizes on mental health may be greater for younger people than for older people.

In this study, a stratified analysis by gender and marital status showed a dose–response relationship between household size and SPD in never-married persons, regardless of gender. According to a large-scale population-based study consisting of cancer-free volunteers aged 30–70 years in Taiwan (9), the prevalence of psychiatric morbidity was significantly higher among those living alone than among those living with their families. However, the association between living alone and psychiatric morbidity was significant in married subjects, but not in unmarried subjects. This study (9) evaluated only the presence or absence of living alone for household size, failed to conduct stratified analyses by gender, and did not use a randomly selected population, which may have contradicted our findings. Protective effects of living with a partner on mental health are considered to be that people living with a partner, whether married or in a common law relationship, may receive social and psychological support from their partners and that sharing financial resources reduces the stress associated with financial problems (38). According to a nationally representative study in Finland targeting the working population (39), women reported more psychological distress than men, but the association between work-related factors and psychological distress was mostly similar among men and women. Furthermore, this previous study (39) suggests that larger households may be exposed to more contradictions between work and home than smaller households, but may also have positive effects on mental health through more social support and work-to-family enrichment. Our findings showed an association between smaller household size and more common SPD among younger and never-married persons, regardless of gender, suggesting that the association between household size and mental health may be influenced by age and marital status, rather than gender difference.

On the other hand, it has been pointed out that in Japan, the mental health of married women may be worse than that of unmarried women, because women are primarily responsible for housework and caring for their household members, which puts a heavy emotional burden on married women (13, 40). In this study, a stratified analysis by age and gender showed that among those aged 60–74, women with two to four household members were significantly more likely to complain of SPD than those with five or more household members, while those living alone had no higher prevalence of SPD. Previous research in Japan has reported that middle-aged women with spouses tend to prioritize their husbands over their own social participation, which may prevent them from obtaining the positive effects of social participation on their mental health (40). Our results suggest that women aged 60–74 are frustrated with the heavy burden of caring for their household members, especially their husbands, which may be damaging their mental health.

This study has some strengths. First, we used the latest data from a large-scale nationwide survey with a high recovery percentage. This ensured sufficient power of the study and the generalizability of this study’s results, and enabled stratified analyses by age, gender, and marital status. Second, we observed a dose–response relationship between household size and SPD; not only living alone but also living in small-member households were associated with a higher prevalence of SPD. Third, because the CSLC collected basic information on socioeconomic status, we could use sufficient covariates.

This study has some limitations. First, because this is a cross-sectional study, the causal relationship between household size and SPD is unclear. That is, this study cannot establish whether small household size causes SPD, or whether people are living alone or in small households due to SPD. Future prospective cohort studies are needed to verify whether individuals living in smaller households have more new-onset SPD than those living in larger households. Second, the global spread of COVID-19 has highlighted the disadvantages of social isolation. Regarding the impact of COVID-19 lockdown on mental health, it has been reported that living alone is a risk factor and living with a partner is a protective factor (41). However, the survey period of this study was before the spread of COVID-19, and the impact of COVID-19 could not be evaluated. In the future, it will be necessary to conduct comparative studies before and after the COVID-19 pandemic, and identify populations whose mental health is likely to be affected by the spread of emerging infectious diseases. Third, although this study adjusted for socio-economic status, smoking status, and illness under treatment, which are important confounding factors, the possibility of unmeasured confounding cannot be denied. For example, social activity is known to reduce the negative association between household size and mental health (11, 40). In this study, employment status (i.e., participation in labor activities) was included as a covariate, but participation in community activities and interactions with friends and acquaintances were not taken into account.

In conclusions, a dose–response relationship indicating the smaller the household size, the greater the number of people with SPD, was observed in younger people and in never-married persons, regardless of gender. The results of this study have revealed that we need to focus not only on older people, but also on younger people, and that we need to focus on small-member households as well as single-person households. Our findings suggest that measures should be considered to ensure younger people and the never-married, who are more vulnerable to adverse mental health effects of small-member households, are able to connect with social support and a local community.

## Data availability statement

The original contributions presented in the study are included in the article/Supplementary material, further inquiries can be directed to the corresponding author.

## Ethics statement

The studies involving humans were approved by Nara Medical University Ethics Committee. The studies were conducted in accordance with the local legislation and institutional requirements. Written informed consent for participation was not required from the participants or the participants’ legal guardians/next of kin in accordance with the national legislation and institutional requirements.

## Author contributions

KT: Conceptualization, Data curation, Formal analysis, Funding acquisition, Writing – original draft, Methodology, Visualization. MS: Project administration, Writing – review & editing. KS: Supervision, Conceptualization, Methodology, Writing – original draft.
