# Supplementary material for: Smaller household size and higher prevalence of serious psychological distress in younger people and never-married people: a nationwide cross-sectional survey in Japan
Source: Front Public Health. 2024 Mar 11;12:1292371. doi: 10.3389/fpubh.2024.1292371 (PMC10962682; doi:10.3389/fpubh.2024.1292371)
Supplement: Supplementary file 1 [file Data_Sheet_1.docx]

**Supplementary Text 1.** **Detailed explanations of the Comprehensive Survey of Living Conditions (CSLC)**

The purpose of the CSLC is to investigate core issues affecting people's lives and to obtain the basic data necessary for the planning and operation of health, labor and welfare administration. The CSLC has been conducted every year since 1986, but large-scale surveys on mental health, smoking, and other health issues are conducted every three years. The survey method was reported as follows (1). The sampling method was a stratified cluster sampling with the census enumeration district as the sampling unit. The survey targets were all households in 5,530 districts randomly selected from approximately 1,040,000 districts based on the 2015 National Census. The 2019 survey targeted 301,334 households, collected 218,332 households (72.5% response rate), and analyzed 217,179 households (72.1% of surveyed households). Although the exact number of household members surveyed was unknown, it was estimated to be about 720,000, and the number of people that could be analyzed was 535,619 (74.4% of the estimated household members surveyed).

**Supplementary Text 2.** **Detailed explanations of covariates**

In an analysis targeting people of all ages, age was classified into 4 groups (20 to 39 years, 40 to 59 years, 60 to 74 years, and 75 years or older) and used as a categorical variable. In a stratified analysis by age, we used a categorical variable classified in every 10-year of age. Marital status was categorized into married, never-married, and widowed/divorced. Education (years of schooling) was categorized into junior high school (≤ 9 years), high school (10–12 years), junior college (13–15 years), and college or higher (≥ 16 years). Equivalent household expenditures (EHE) per month were divided into three categories by tertiles: low (< 106 thousand Japanese yen), middle (106–155 thousand Japanese yen), and high (> 155 thousand Japanese yen). Housing tenure was dichotomized into owner-occupiers and renters. Smoking status was categorized into current smokers, ex-smokers, and never-smokers. Regarding employment contract, a previous study examined the association of occupation, employment contract, and company size with mental health among Japanese employees, and found that only employment contract was associated with poor mental health, suggesting that employment contract may be an important indicator for Japanese social class (2). Employment contract was categorized into regular employees, non-regular employees (e.g., part-time employees, temporary employees, and contract employees), self-employed, and non-working. Regarding illness under treatment, diabetes mellitus, hypertension, stroke, myocardial infarction, and cancer have been reported to be associated with serious psychological distress (SPD) in Japanese community residents aged 40 and over (3). Therefore, we defined these five diseases as chronic medical conditions (CMC), and divided study participants into three groups: people with CMC, those with illness other than CMC, and those without illness under treatment.

**References**

1. Ministry of Health, Labour and Welfare of Japan. Comprehensive Survey of Living Conditions. Survey overview. (2023). <https://www.mhlw.go.jp/toukei/list/20-21tyousa.html#anchor02>. [ Accessed September 9, 2023]
2. Inoue A, Kawakami N, Tsuchiya M, Sakurai K, Hashimoto H. Association of occupation, employment contract, and company size with mental health in a national representative sample of employees in Japan. J Occup Health. (2010) 52:227–240.
3. Kuriyama S, Nakaya N, Ohmori-Matsuda K, et al. Factors associated with psychological distress in a community-dwelling Japanese population: the Ohsaki Cohort 2006 Study. J Epidemiol. (2009) 19:294–302.

**
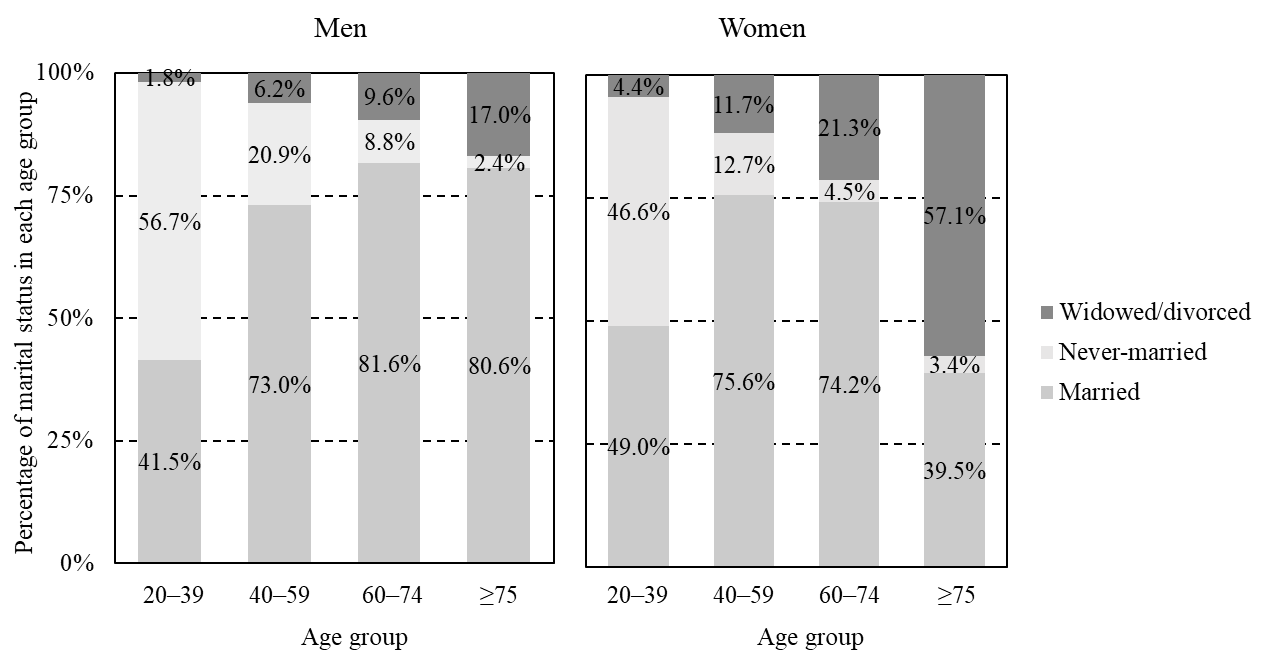
**

**Supplementary Figure 1. Marital status by age and gender**

**Supplementary Table 1. Question about household status**

Please answer the current status of your household as of June 2, 2022.

- A household is a group of people (household members) who usually live together and share living expenses.
- Household members include those who are away from home temporarily (up to 3 months) due to travel or business-related trips, those who are effectively living as a married couple but have not submitted a marriage registration, and people who have no kin relationship.
- On the other hand, household members exclude those who are away from the household due to their work or for their education, and those who are in social welfare facilities.


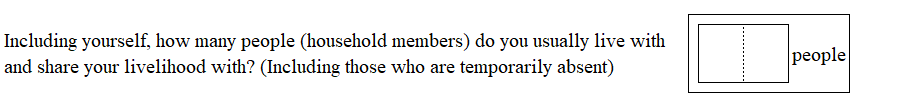


**Supplementary Table 2.** **The Kessler 6-item Psychological Distress Scale (K6)**

The following questions ask about how you have been feeling during the **past 30 days**.

For each question, please circle the number that best describes how often you had this feeling.

| **Q1.** | **During the past 30 days, about**  **how often did you feel …** | **All of  the time** | **Most of  the time** | **Some of  the time** | **A little of  the time** | **None of  the time** |
| --- | --- | --- | --- | --- | --- | --- |
| **a.** ... | nervous? | **1** | **2** | **3** | **4** | **5** |
| **b.** ... | hopeless? | **1** | **2** | **3** | **4** | **5** |
| **c.** ... | restless or fidgety? | **1** | **2** | **3** | **4** | **5** |
| **d.** ... | so depressed that nothing could  cheer you up? | **1** | **2** | **3** | **4** | **5** |
| **e.** ... | that everything was an effort? | **1** | **2** | **3** | **4** | **5** |
| **f.** ... | worthless? | **1** | **2** | **3** | **4** | **5** |

Responses are given on a 5-point scale ranging from "all of the time" to "none of the time" and are scored on a scale of 0 to 4.

The total score ranges from 0 to 24, and the higher scores indicate a high level of psychological stress.

The K6 is available for download on the National Comorbidity Survey website. <https://www.hcp.med.harvard.edu/ncs/k6_scales.php>. [Accessed February 14, 2024].

Kessler RC, Barker PR, Colpe LJ, Epstein JF, Gfroerer JC, Hiripi E, et al. Screening for serious mental illness in the general population. Arc Gen Psychiatry. (2003) 60:184–189.

Copyright © Ronald C. Kessler, PhD. All rights reserved.

**Supplementary Table 3. Adjusted odds ratio for serious psychological distress by gender**

| Variable | Category | Men (n = 193,346) | |  | Women (n = 212,214) | |
| --- | --- | --- | --- | --- | --- | --- |
|  | classification | N | AOR (95% CI) |  | N | AOR (95% CI) |
| Household size | 5 or more | 26,099 | 1.00 |  | 28,920 | 1.00 |
|  | 3 or 4 | 84,962 | 1.05 (0.97–1.14) |  | 91,225 | 0.99 (0.93–1.06) |
|  | Two | 58,435 | 1.09 (0.998–1.19) |  | 65,199 | 1.05 (0.98–1.13) |
|  | One (living alone) | 23,850 | 1.37 (1.23–1.52)^**^ |  | 26,870 | 1.03 (0.94–1.13) |
|  |  |  | *P* for trend < 0.001 |  |  | *P* for trend = 0.146 |
| Marital status | Married | 134,346 | 1.00 |  | 133,712 | 1.00 |
|  | Never-married | 44,110 | 1.31 (1.23–1.40)^**^ |  | 33,506 | 1.65 (1.56–1.75)^**^ |
|  | Widowed/divorced | 14,890 | 1.46 (1.33–1.60)^**^ |  | 44,996 | 1.39 (1.31–1.48)^**^ |
| Age | Aged 20–39 | 43,224 | 1.00 |  | 44,196 | 1.00 |
|  | Aged 40–59 | 67,471 | 0.73 (0.68–0.78)^**^ |  | 70,532 | 0.74 (0.70–0.79)^**^ |
|  | Aged 60–74 | 54,771 | 0.22 (0.20–0.24)^**^ |  | 58,253 | 0.25 (0.23–0.28)^**^ |
|  | Aged 75 or older | 27,880 | 0.22 (0.19–0.24)^**^ |  | 39,233 | 0.29 (0.26–0.32)^**^ |
| Education  (years of  schooling) | < 10 years | 20,012 | 1.00 |  | 24,992 | 1.00 |
|  | 10–12 years | 72,218 | 0.81 (0.74–0.88)^**^ |  | 80,077 | 0.80 (0.75–0.86)^**^ |
|  | 13–15 years | 20,162 | 0.90 (0.81–0.9997)^*^ |  | 48,209 | 0.70 (0.64–0.75)^**^ |
|  | > 15 years | 53,569 | 0.78 (0.71–0.86)^**^ |  | 28,018 | 0.72 (0.65–0.79)^**^ |
|  | Missing | 27,385 | 0.89 (0.81–0.99)^*^ |  | 30,918 | 0.79 (0.73–0.87)^**^ |
| EHE (Thousand  Japanese yen  per month) | Lower tertile (< 106) | 60,957 | 1.00 |  | 69,487 | 1.00 |
|  | Middle tertile (106–155) | 59,243 | 1.00 (0.94–1.06) |  | 65,518 | 0.98 (0.93–1.03) |
|  | Upper tertile (> 155) | 62,054 | 0.96 (0.90–1.02) |  | 65,426 | 0.99 (0.94–1.05) |
|  | Missing | 11,092 | 1.02 (0.90–1.14) |  | 11,783 | 1.02 (0.92–1.12) |
| Employment  contract | Regular employees | 84,595 | 1.00 |  | 43,859 | 1.00 |
|  | Non-regular employees | 30,055 | 1.12 (1.04–1.21)^*^ |  | 54,101 | 1.13 (1.06–1.21)^**^ |
|  | Self-employed | 24,398 | 0.92 (0.84–1.01) |  | 16,829 | 1.06 (0.96–1.17) |
|  | Non-working | 47,525 | 2.33 (2.16–2.50)^**^ |  | 92,597 | 1.76 (1.66–1.87)^**^ |
|  | Missing | 6,773 | 1.52 (1.31–1.76)^**^ |  | 4,828 | 1.54 (1.34–1.78)^**^ |
| Housing  tenure | Owner-occupiers | 152,421 | 1.00 |  | 168,705 | 1.00 |
|  | Renters | 40,925 | 1.31 (1.23–1.39)^**^ |  | 43,509 | 1.32 (1.26–1.39)^**^ |
| Smoking  status | Never | 116,255 | 1.00 |  | 187,088 | 1.00 |
|  | Former | 17,142 | 1.11 (1.01–1.21)^*^ |  | 4,962 | 1.57 (1.41–1.75)^**^ |
|  | Current | 57,603 | 1.08 (1.02–1.14)^*^ |  | 17,944 | 1.69 (1.59–1.80)^**^ |
|  | Missing | 2,346 | 1.23 (0.98–1.54) |  | 2,220 | 1.15 (0.93–1.43) |
| Illness under  treatment | Absent | 104,421 | 1.00 |  | 107,382 | 1.00 |
|  | Illness other than CMC | 43,252 | 2.48 (2.34–2.62)^**^ |  | 61,821 | 2.71 (2.59–2.84)^**^ |
|  | CMC | 39,510 | 1.43 (1.33–1.55)^**^ |  | 35,321 | 1.60 (1.49–1.72)^**^ |
|  | Missing | 6,163 | 2.07 (1.81–2.37)^**^ |  | 7,690 | 2.19 (1.96–2.45)^**^ |

AOR, adjusted odds ratio; CI, confidence interval; CMC, chronic medical conditions; EHE, Equivalent household expenditures. ^*^*P* < 0.05, ^**^*P* < 0.001. AOR was adjusted for household size, marital status, age, education, EHE, employment contract, housing tenure, smoking status, and illness under treatment.
